# Pharmacokinetics of a Single Intake of a Self-Emulsifying Drug Delivery System Containing the Triglyceride Form of DHA: A Randomized, Double-Blinded, Crossover Study

**DOI:** 10.1093/cdn/nzac122

**Published:** 2022-07-25

**Authors:** Mariko Hayashida, Yuri Ishii, Tomoki Adachi, Rie Imai, Nobuo Uotsu, Kei Yui

**Affiliations:** Research Institute, FANCL Corporation, Yokohama, Japan; Research Institute, FANCL Corporation, Yokohama, Japan; Research Institute, FANCL Corporation, Yokohama, Japan; Research Institute, FANCL Corporation, Yokohama, Japan; Research Institute, FANCL Corporation, Yokohama, Japan; Research Institute, FANCL Corporation, Yokohama, Japan

**Keywords:** fish oils, omega-3, docosahexaenoic acid, eicosapentaenoic acid, bioavailability, absorption, self-emulsifying delivery system, drug delivery systems, clinical trial, randomized controlled trial

## Abstract

**Background:**

The health benefits of n–3 (ω-3) PUFAs are well studied. A self-emulsifying drug delivery system (SEDDS) is expected to improve n–3 PUFA absorption.

**Objectives:**

The present study investigated how a single ingestion of a new SEDDS containing the triglyceride (TG) form of DHA (22:6n–3) (DHA/TG) would affect the plasma DHA concentration in healthy participants.

**Methods:**

Fifteen healthy participants (age: 20–65 y; BMI: 18.5–25 kg/m^2^) were enrolled in this randomized, double-blind, crossover study. Participants in a fasting state consumed a single dose of 920 mg DHA and 80 mg EPA (20:5n–3) in SEDDS soft capsules (SEDDS capsule) or non-emulsifying soft capsules (control capsule). Blood was sampled at 0, 1.5, 3, 5, 7, and 9 h after dosing. The primary outcome was the baseline-adjusted incremental AUC (iAUC) for plasma DHA concentrations (iAUC_DHA).

**Results:**

The iAUC_DHA was significantly higher for the SEDDS capsule (147.9 ± 15.8 µg·h/mL) than for the control capsule (106.4 ± 18.1 µg·h/mL) (*P* = 0.018; SEDDS/control ratio: 1.4:1). However, plasma EPA concentrations and iAUC values did not significantly differ between the SEDDS and control capsules. Cmax was significantly higher with the SEDDS capsule for both DHA (*P* = 0.019) and EPA (*P* = 0.012) than with the control capsule.

**Conclusions:**

These results suggest that a SEDDS improves the absorbability of DHA/TG in healthy participants. This indicates that SEDDS capsules would be beneficial for efficient ingestion of DHA.

This trial was registered at https://www.umin.ac.jp/ctr/ as UMIN000044188.

## Teaser

This clinical study showed that a self-emulsifying drug delivery system improves the bioavailability of the triglyceride form of docosahexaenoic acid in healthy participants.

## Introduction

DHA (22:6n–3) is an n–3 PUFA with 22 carbon chains and 6 double bonds. Since the first epidemiologic studies by Bang, Dyerberg, and colleagues in the 1970s (1–3), numerous studies have demonstrated that the intake of n–3 fatty acids, particularly DHA and EPA (20:5n–3), is effective for preventing cardiovascular disease in humans. These findings have been supported by multiple meta-analyses (4–7). PUFAs are also expected to have preventive effects on cognitive decline and dementia (8). The Dietary Reference Intakes for Japanese (2020 edition) (9) recommend 1.6–2.2 g/d of n–3 fatty acids, whereas the 2010 edition advocated ≥1.0 g/d of DHA and EPA. However, according to the 2019 National Health and Nutrition Survey, the mean daily DHA and EPA intake among Japanese people is estimated at 0.8 g, which is below the recommended 1.0 g (10). Supplements could be used to make up for this shortage.

DHA and EPA exist as various esters. The raw materials that can be used in supplements in Japan are a triglyceride (TG) form (DHA/TG, EPA/TG) and a phospholipid (PL) form (DHA/PL, EPA/PL). DHA/PL and EPA/PL are known to be more bioavailable than DHA/TG and EPA/TG (11). However, commercially available fish oil, the main source of DHA/TG and EPA/TG, is more cost effective than krill oil, the main source of DHA/PL and EPA/PL (12). Based on the regulatory system of “Foods with Health Claims” in Japan, the efficacy of “Foods with Functional Claims” (FFC) and “Foods for Specified Health Uses” (FOSHU) must be confirmed based on the results of double-blind, comparative studies in humans. Most of the DHA/EPA-containing foods registered as FFC or FOSHU are of the TG form, which are supplied from fish oil, accounting for 70% of the items in the 2021 registration. Therefore, improving the absorption of DHA/TG and EPA/TG is still useful even at present. A highly purified ethyl ester (EE) type (DHA/EE, EPA/EE) is used in pharmaceuticals. From the perspective of dosage and administration, absorption of this type is poor in the fasting state. But it improves if taken after a high-fat meal (13–16); therefore, patients are instructed to take it orally after meals. DHA/TG and EPA/TG have also been reported to be affected by the diet (16); however, because the timing of intake is not specified for supplements like it is for pharmaceuticals, it would be helpful to modify the formulation to lessen the impact of factors such as whether it is taken with food and the amount of fat intake.

Self-emulsifying drug delivery systems (SEDDSs) are lipid-based formulations composed of a mixture of oil and surfactants, and optionally cosurfactants and cosolvents, that in contact with an aqueous phase, such as a digestive liquid, and under mild agitation simulating the movements of the gastrointestinal tract, will form a fine and stable emulsion (17, 18). Improved absorbability of DHA/EE and EPA/EE through the use of SEDDS has been reported (19–23). In contrast, research on improving absorption with emulsified DHA/TG and EPA/TG has mainly focused on comparing gelled or emulsified-gelled foods with non-emulsified soft capsules (24–26). Although an open-label study of capsule formats reported improved absorption of EPA, there was no significant improvement in DHA absorption (27). Thus, further research is needed on this topic. Fish oil, the raw material for DHA/TG, has a peculiar odor and, therefore, the capsule format is considered useful for continuous intake in adults. In this study, we developed a SEDDS soft capsule containing DHA/TG and examined absorbability in comparison with a regular DHA/TG preparation in a randomized, double-blind, crossover study.

## Methods

### Ethics considerations

This study was conducted after review and approval by the clinical research ethics committee of the FANCL Research Institute (application no. C2020-005, approved 11 May, 2021). The study was conducted in accordance with the principles of the Declaration of Helsinki (adopted June 1964, revised October 2013). Protection of the subjects’ human rights was given due consideration and the ethical guidelines for medical and health research involving human subjects (2014 Ministry of Education, Culture, Sports, Science and Technology notice no. 3, revised 28 February, 2017) were followed. The study was conducted at the FANCL Research Institute in June and July 2021. Before the start of the study, it was registered with the University Hospital Medical Information Network Clinical Trials Registry (UMIN-CTR), which is operated by the University Hospital Medical Information Network Center (ID: UMIN000044188; https://center6.umin.ac.jp/cgi-open-bin/ctr_e/ctr_view.cgi?recptno=R000050425).

### Participants

Sample size was determined before recruitment, based on anticipated access to participants, and recruitment ceased when we reached our goal of 15 usable participants. The following data were collected from candidate study participants: sex, age, health check results, medical history, gastrointestinal symptoms, current medications, health foods consumed, dietary habits, smoking habit, alcohol consumption, and allergies. We also checked their background, health status, height and weight, and vital signs (blood pressure, pulse). Fifteen candidate participants who met the inclusion criteria and did not meet the exclusion criteria were included in the study. The inclusion criteria were age between 20 and 65 y at the time of consent, a healthy male or female, and BMI (in kg/m^2^) between 18.5 and 25.0. The exclusion criteria were *1*) participants with dyslipidemia (TG ≥ 150 mg/dL and/or LDL cholesterol ≥ 140 mg/dL and/or HDL cholesterol < 40 mg/dL; defined by the Japanese Society for Arteriosclerosis Prevention Guidelines) (28); *2*) participants with severe liver diseases, digestive organ diseases, kidney diseases, and heart diseases; *3*) participants who had a history of surgical resection of the gastrointestinal tract (except for appendectomy); *4*) participants who had various symptoms of indigestion; *5*) participants who were bleeding or in danger of bleeding; *6*) participants who couldn't stop taking a supplement or medicine including DHA and EPA during the study period; *7*) difficulty in collecting blood (participants for whom it is not acceptable to have multiple blood draws in 1 d; participants who are at high risk of internal bleeding, such as those with small, deep blood vessels; etc.); *8*) smokers; *9*) participants who planned to participate in another clinical study; *10*) participants who intended to become pregnant or were lactating; and *11*) participants who were judged as unsuitable for the study by the investigator for another reason.

### Trial supplements

The trial supplements were 2 types of soft capsules: One with fish oil containing DHA/TG, an emulsifier (glycerate), olive leaf extract, and antioxidants (catechin and vitamin E) (manufactured by FANCL Corporation), and the other with only fish oil containing DHA and antioxidants (vitamin E and catechin). The former spontaneously dispersed and formed a stable emulsion in “2nd Fluid for dissolution test” at 37°C, confirming it to have a self-emulsifying ability (it is hereinafter referred to as the SEDDS capsule). “2nd Fluid for dissolution test” is a simulated intestinal fluid as defined in the 18th revised Japanese Pharmacopoeia (29): a mixture of phosphate buffer solution (pH: 6.8) and water (1:1). Whereas, the latter was confirmed not to have a self-emulsifying action as it formed an oil layer at the interface, without dispersing (it is hereinafter referred to as the control capsule). Olive leaf extract may contain emulsifying substances. It was not included in the control capsule to clarify the self-emulsifying effect of the SEDDS capsule. Both types of capsules contained 92 mg DHA and 8 mg EPA per capsule and had a caramel-colored coating, so they were visually indistinguishable. The intake for both trial supplements was 10 capsules (DHA: 920 mg; EPA: 80 mg) per dose. The capsule size (oval, 382 mg/capsule) of the trial supplement was just small enough to be taken 10 capsules at 1 time, confirming that it is acceptable for a healthy adult to consume.

### Procedure

This was a randomized, double-blind, crossover study. The consenting participants were assigned to 1 of 2 sequences (sequence A, sequence B) according to an allocation table created to generate random sequences. In phase I of the study, sequence A was given the SEDDS capsule and sequence B was given the control capsule. After a washout period of ≥1 wk, sequence A was given the control capsule and sequence B was given the SEDDS capsule (phase II). Because the trial supplements were ingested on a single occasion, compliance was visually confirmed by the researcher. The allocation table had been prepared by people who were not involved in the study and the key was opened after data collection and analysis were completed. Everyone involved in the study, including the study participants, investigators, and staff, were blinded to allocation.

**FIGURE 1:**
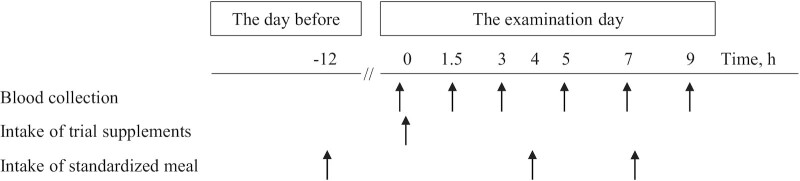
Time schedule of the study (phase I and phase II). Blood samples were collected at 0, 1.5, 3, 5, 7, and 9 h after dosing. Participants consumed a single dose of SEDDS soft capsules containing DHA/TG (SEDDS capsule) or non-emulsifying soft capsules containing DHA/TG (control capsule) in a fasting state. In both treatments, the single dose contained 920 mg DHA and 80 mg EPA. The study participants consumed standardized meals at 12 h before dosing, 4 h after dosing, and after blood sampling at 7 h after dosing. SEDDS, self-emulsifying drug delivery system; TG, triglyceride.

The participants were prohibited from consuming any drugs or health foods containing DHA or EPA from the time they gave consent until the end of the study. In addition, the consumption of fish and shellfish was prohibited from 1 wk before the examinations, and dietary records were kept. The day before each examination, a standardized meal (rice, simmered chicken and root vegetables, boiled pumpkin, soup, jelly: 748 kcal, 13.8 g protein, 3.5 g fat, 167.9 g carbohydrates) was consumed sometime between 18:00 and 21:00, after which only water consumption was freely allowed. On the day of the examination, the participants gathered at 09:00 while still fasting. After an interview and measuring their height, body weight, BMI, blood pressure, and pulse, a blood sample was collected (baseline). Then, 10 capsules of the trial supplement were ingested with 200 mL of water. Blood samples were collected after 1.5, 3, 5, 7, and 9 h. Participants consumed a standardized lunch (rice, vegetable curry, soup, yogurt: 492 kcal, 17.9 g protein, 0.8 g fat, 100.3 g carbohydrates) 4 h after ingestion of the supplement and a standardized snack (jelly drink: 180 kcal, 0.0 g protein, 0.0 g fat, 45.0 g carbohydrates) after the 7 h blood sampling (**Figure 1**).

The blood samples were lightly mixed 4–5 times at room temperature, centrifuged at 1000 × *g* at room temperature for 10 min, and stored at −28 °C until analysis. Plasma DHA and EPA concentrations were measured at SRL Inc., using GC (GC-2010, Shimadzu Corp.).

### Outcomes

The primary outcome was the baseline-adjusted incremental AUC (iAUC) for plasma DHA concentrations (iAUC_DHA). Baseline was defined as the predose blood collection time point in each test period. The secondary outcomes were the AUC for the plasma DHA concentration (AUC_DHA), the AUC for the plasma EPA concentration (AUC_EPA), the baseline-adjusted iAUC for plasma EPA concentrations (iAUC_EPA), baseline-adjusted plasma DHA and EPA concentrations (ΔDHA, ΔEPA), peak plasma DHA and EPA concentrations (Cmax), and the time to reach peak concentrations (Tmax). The AUC was calculated using the linear trapezoidal rule and the iAUC was calculated ignoring the area below the baseline, as described by Brouns et al. (30).

Participants were monitored and asked to report any adverse events experienced while at the research center for safety analysis.

### Statistical analysis

Statistical analysis was performed on data from participants who completed the entire research schedule and who had confirmed compliance. Their compliance during the study period was checked by dietary records and interviews. The background characteristics of sequences A and B were compared using a Student's *t* test or Pearson's chi-square test. The order effect was evaluated to confirm the carryover effect of the primary outcome, iAUC_DHA. After that, food effect was evaluated using ANOVA adjusted for period effect. The secondary outcomes were evaluated in the same way as the primary outcome. JMP® 14.1.0 (SAS Institute Inc.) and Microsoft Excel 2013 (Microsoft) were used for the statistical analyses. Data are expressed as mean ± SD or SE, and the significance level was 5%, 2-tailed.

## Results

### Participant characteristics

**FIGURE 2:**
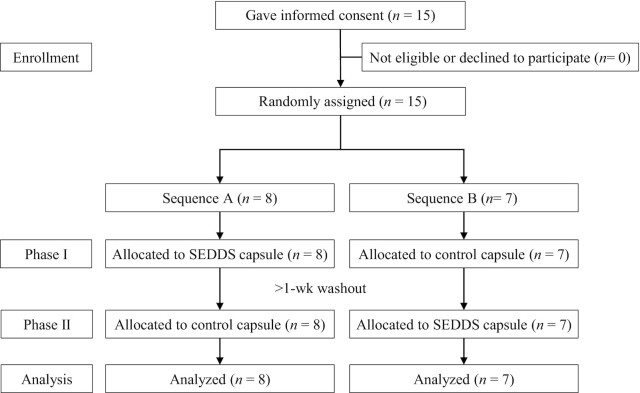
Participant flow diagram over the study period. SEDDS, self-emulsifying drug delivery system.

Background surveys on the 15 people who gave consent confirmed that they met the inclusion criteria and did not meet any of the exclusion criteria. The 15 participants were assigned to sequence A (*n* = 8) or sequence B (*n* = 7). Because all participants completed the trial, the analysis set comprised 15 participants. **Figure 2** shows a flow diagram of the trial and **Table 1** shows the participants’ background characteristics. The plasma DHA and plasma EPA values are fasting concentrations. There were no significant differences between the sequences in any of these characteristics, including plasma DHA (*P* = 0.0501). Sequence B had 4 males and 2 females, but there was no significant difference between sequence A and sequence B as assessed using Pearson's chi-square test.

**TABLE 1:** Demographic characteristics of the study participants^1^

	All (*n* = 15)	Sequence A (*n* = 8)	Sequence B (*n* = 7)	*P*
Age, y	35.9 ± 5.7	35.8 ± 7.6	36.1 ± 3.2	0.901
Sex, male/female	9/6	4/4	5/2	0.398
BMI, kg/m^2^	21.50 ± 2.32	20.56 ± 2.05	22.57 ± 2.26	0.094
SBP, mm Hg	108.6 ± 12.4	107.1 ± 9.8	110.3 ± 15.4	0.639
DBP, mm Hg	68.9 ± 9.9	68.1 ± 7.7	69.7 ± 12.5	0.768
Pulse, bpm	69.5 ± 8.0	70.8 ± 7.9	68.1 ± 8.5	0.584
Plasma DHA, µg/mL	78.90 ± 20.51	69.38 ± 7.33	89.79 ± 25.69	0.0501
Plasma EPA, µg/mL	17.01 ± 9.16	15.86 ± 9.25	18.31 ± 9.61	0.623

1 Values are mean ± SD unless otherwise indicated. For *P* values, only sex was assessed using Pearson's chi-square test; other parameters were assessed using Student's *t* test. bpm, beats per minute; DBP, diastolic blood pressure; SBP, systolic blood pressure.

### Blood parameters

The carryover effect was evaluated for the primary outcome of iAUC_DHA; the order effect (*P* = 0.735) was nonsignificant. Therefore, we considered the crossover design appropriate for this study.

**FIGURE 3:**
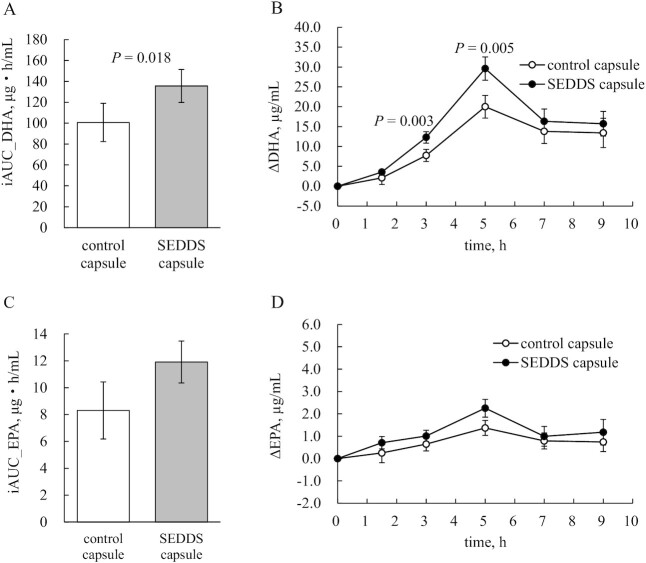
iAUCs and mean plasma concentrations after ingestion of the capsules. (A, C) iAUCs at time 0–9 h [(A) iAUC_DHA, (C) iAUC_EPA)] after administration of the control capsule or SEDDS capsule. Data are mean ± SE. (B, D) Mean plasma concentrations of (B) ΔDHA and (D) ΔEPA at 0–9 h after ingestion of the control capsule or SEDDS capsule. Data are mean ± SE. iAUC, incremental area under the curve; SEDDS, self-emulsifying drug delivery system.

**Figure 3** shows ΔDHA and ΔEPA at each time point, and the iAUC values. The primary outcome iAUC_DHA (Figure 3A) was significantly higher for the SEDDS capsule (147.9 ± 15.8 μg·h/mL) than for the control capsule (106.4 ± 18.1 μg·h/mL) (*P* = 0.018). There was no significant difference in the period effect (*P* = 0.358). ΔDHA (Figure 3B) was significantly higher for the SEDDS capsule at 3 h (SEDDS capsule: 12.3 ± 1.4 μg/mL; control capsule: 7.7 ± 1.5 μg/mL; *P* = 0.003) and 5 h (SEDDS capsule: 29.6 ± 2.9 μg/mL; control capsule: 20.0 ± 2.8 μg/mL; *P* = 0.005) after ingestion. iAUC_EPA (Figure 3C) and ΔEPA (Figure 3D) were higher with the SEDDS capsule, albeit not significantly. **Table 2** shows the plasma DHA and EPA concentrations at each time point, AUC, Tmax, and Cmax. Plasma DHA was significantly higher with the SEDDS capsule than with the control capsule 5 h after ingestion (*P* = 0.011). Blood EPA concentrations were significantly higher with the SEDDS capsule 3 h (*P* = 0.033), 5 h (*P* = 0.016), and 9 h (*P* = 0.043) after ingestion. There was no significant difference in AUC_DHA between the test supplements (*P* = 0.102), although AUC_EPA was significantly higher with the SEDDS capsule than with the control capsule (*P* = 0.032). Cmax was significantly higher with the SEDDS capsule for both DHA (*P* = 0.019) and EPA (*P* = 0.012). Tmax for DHA and EPA did not significantly differ between the trial supplements.

**TABLE 2:** Pharmacokinetic parameters for plasma DHA and EPA concentrations per trial supplement^1^

	Control capsule (*n* = 15)	SEDDS capsule (*n* = 15)	*P*
Plasma DHA			
0 h, µg/mL	79.87 ± 5.41	80.00 ± 5.36	0.901
1.5 h, µg/mL	81.98 ± 5.10	83.53 ± 5.33	0.482
3 h, µg/mL	87.59 ± 5.71	92.27 ± 5.72	0.073
5 h, µg/mL	99.86 ± 6.84	109.58 ± 6.63	0.011*
7 h, µg/mL	93.68 ± 6.39	96.33 ± 5.88	0.378
9 h, µg/mL	93.27 ± 6.45	95.70 ± 5.50	0.379
AUC, µg · h/mL	816.51 ± 53.53	854.29 ± 50.90	0.102
Tmax, h	5.5 ± 0.4	5.0 ± 0.0	0.234
Cmax, µg/mL	100.47 ± 6.94	109.58 ± 6.63	0.019*
Plasma EPA			
0 h, µg/mL	17.59 ± 2.53	18.88 ± 2.69	0.060
1.5 h, µg/mL	17.84 ± 2.62	19.59 ± 2.88	0.077
3 h, µg/mL	18.24 ± 2.66	19.89 ± 2.74	0.033*
5 h, µg/mL	18.96 ± 2.68	21.13 ± 2.56	0.016*
7 h, µg/mL	18.38 ± 2.59	19.87 ± 2.50	0.081
9 h, µg/mL	18.33 ± 2.60	20.05 ± 2.52	0.043*
AUC, µg · h/mL	164.89 ± 23.56	180.41 ± 23.64	0.032*
Tmax, h	5.5 ± 0.7	5.2 ± 0.5	0.629
Cmax, µg/mL	19.33 ± 2.65	21.82 ± 2.77	0.012*

1 Values are mean ± SE unless otherwise indicated. *P* values were assessed using ANOVA. Cmax, peak concentration; SEDDS, self-emulsifying drug delivery system; Tmax, time to reach peak concentration; *, *P* < 0.05.

### Safety

Safety was examined in the 15 participants who ingested the trial supplements at least once. No adverse events associated with the consumption of the trial supplements were observed.

## Discussion

We investigated the usefulness of SEDDS capsules for DHA intake in healthy Japanese adults in a randomized, double-blind, crossover study. The results showed that each of iAUC_DHA, AUC_EPA, and both DHA and EPA Cmax were significantly higher for the SEDDS capsule than for the control capsule, suggesting that the SEDDS capsule was superior to the control capsule in DHA/TG and EPA/TG absorption. Previous studies on DHA/TG self-emulsifying formulations revealed improved absorption of DHA in liquid and gel formulations (24–26), and although a capsule-type formulation showed improved EPA absorption, there was no significant improvement in DHA absorption (27). The present study demonstrated improved absorption from a soft-capsule SEDDS, which is important because SEDDSs are more convenient than regular foods from the perspective of compliance.

Orally ingested lipids (TG) are hydrolyzed by lipase at the sn-1 and sn-3 positions to produce fatty acids and 2-monoacylglycerol (2-MG). Bile salts in the bile incorporate the lipids to form micelles, making them easier to digest and absorb. 2-MG absorbed in the intestinal tract is reconstituted into TG through the action of long-chain fatty acids and monoacylglycerol-acyltransferase in the intestinal cells and is then transported to the blood. In this absorption mechanism, micelle formation is crucial for DHA/TG digestion and absorption. The SEDDS capsule spontaneously formed micelles in artificial intestinal fluid, whereas the control capsule did not disperse or form micelles. In other words, the SEDDS capsule spontaneously emulsified when in contact with intestinal juice, even in a fasting state, in which little bile acid is being secreted, which was demonstrated to facilitate the action of lipases to yield better absorption than the control capsule.

DHA/EE and EPA/EE are widely used around the world as drugs and supplements because of their ease of concentration to high purity. Therefore, research on improving absorption via SEDDS for DHA and EPA products has mainly focused on DHA/EE and EPA/EE (19–23). In Japan, from the viewpoint of legal regulations, DHA/EE and EPA/EE are used only as pharmaceutical products and these products are not provided as food as supplements are. DHA/TG, EPA/TG, DHA/PL, and EPA/PL are used as supplements in Japan. In particular, DHA/TG is still widely used because of its superior cost performance (12).

DHA/EE and EPA/EE have a lower absorption rate in certain conditions, such as low fat intake, than DHA/TG and EPA/TG (31–34). Beckermann et al. (33) compared blood plasma concentrations ≤36 h after single ingestion of capsules containing DHA/EE, EPA/EE or DHA/TG, EPA/TG in participants consuming a low-fat diet. They found that the iAUC for DHA/EE was 47.5% of that for DHA/TG. This difference in absorption is suggested to be due to differences in the decomposition efficiency of lipase and the efficiency of TG re-esterification (difference in 2-MG supply) (34–36). It has been reported that the iAUC of DHA/EE, which has low absorption when ingested under a low-fat diet, can be increased by 2- to 5-fold through SEDDS processing (17–21). The present study confirmed that SEDDS processing of DHA/TG, which has relatively good fasting absorption, improved absorption by 1.4-fold, indicating that SEDDS processing is highly significant for DHA/TG. Direct comparisons with findings in previous studies are difficult because of differences in factors such as the intervention dose, timing of measurements, ethnicity, and SEDDS processing method. Nevertheless, in places like Japan that do not allow the use of DHA/EE in foods, we believe the improvement in DHA/TG absorbability demonstrated in the present study is sufficiently beneficial.

Fish and shellfish consumption is increasing worldwide, and per capita annual consumption was estimated to be 20.5 kg in 2018. This is not only due to increased production, but is influenced by various factors such as logistical and other technological developments and heightened consumer awareness of the health benefits of fish (37). However, although consumption in Japan since that year has been higher than the global average at 23–24 kg, Japanese people, particularly younger people, are consuming less fish than before. The annual consumption has declined since peaking at 40.2 kg in 2001 (38, 39). Reduced fish consumption affects DHA/EPA intake, and lack of these essential fatty acids is considered to increase the risk of various diseases, which highlights the importance of efficient supplementation for maintaining good health. Self-emulsifying DHA/TG can be used to supplement those n–3 fatty acids that people tend to be deficient in, regardless of the amount of fat intake in the diet, and can contribute to reducing the risk of various diseases.

This study had some limitations. The target population of this study was limited to Japanese people. Nevertheless, the findings are considered quite generalizable because the population included both men and women with a wide age range. Furthermore, as can be inferred from previous research, values are unlikely to fluctuate much after Tmax and, thus, the results are not expected to be reversed (27). Blood dynamics ≥9 h after ingestion are a topic for future study.

In conclusion, we investigated DHA/TG absorbability from a self-emulsifying soft capsule in comparison with a non-emulsifying soft capsule. The results showed that the rate of DHA absorption was improved with the soft capsule that had a self-emulsifying ability, even when ingested in a fasting state. This indicates that SEDDS capsules would be beneficial for efficient ingestion of DHA.

## Data Availability

Data described in the article will be made available upon request pending application and approval.

## Notes

Supported by FANCL Corporation, which is a cosmetics and health food research and development company. FANCL Corporation funded the study and participated in the study design, implementation, analysis of the data and writing of this article.

Author disclosures: MH, YI, TA, RI, NU and KY were employees of FANCL.

Abbreviations used: Cmax, peak concentration; DHA/EE, ethyl ester form of DHA; DHA/PL, phospholipid form of DHA; DHA/TG, triglyceride form of DHA; EE, ethyl ester; EPA/EE, ethyl ester form of EPA; EPA/PL, phospholipid form of EPA; EPA/TG, triglyceride form of EPA; FFC, Foods with Functional Claims; FOSHU, Foods for Specified Health Uses; iAUC, incremental area under the curve; PL, phospholipid; SEDDS, self-emulsifying drug delivery system; TG, triglyceride; Tmax, time to reach peak concentration; 2-MG, 2-monoacylglycerol.
